# Novel SOI-Biosensor Topology for the Detection of an Acute Myocardial Infarction Marker — Troponin I

**DOI:** 10.17691/stm2024.16.1.04

**Published:** 2024-02-28

**Authors:** A.A. Cheremiskina, V.V. Krasitskaya, V.M. Generalov, L.A. Frank, A.V. Glukhov, M.V. Kruchinina, G.A. Kudrov, D.E. Serdyuk, V.K. Grabezhova

**Affiliations:** Junior Researcher; Federal Budgetary Research Institution, State Research Center of Virology and Biotechnology “Vector”, Federal Service for Surveillance on Consumer Rights Protection and Human Wellbeing, Koltsovo, Novosibirsk Region, 630559, Russia; Senior Researcher; Institute of Biophysics of the Siberian Branch of the Russian Academy of Sciences, Federal Research Center “Krasnoyarsk Science Center of the Siberian Branch of the Russian Academy of Sciences”, 50/50 Bld., Akademgorodok St., Krasnoyarsk, 660036, Russia; Leading Researcher; Federal Budgetary Research Institution, State Research Center of Virology and Biotechnology “Vector”, Federal Service for Surveillance on Consumer Rights Protection and Human Wellbeing, Koltsovo, Novosibirsk Region, 630559, Russia; Professor, Faculty of Automation and Computer Engineering; Novosibirsk State Technical University, 20 Prospekt K. Marksa, Novosibirsk, 630073, Russia; Chief Researcher; Institute of Biophysics of the Siberian Branch of the Russian Academy of Sciences, Federal Research Center “Krasnoyarsk Science Center of the Siberian Branch of the Russian Academy of Sciences”, 50/50 Bld., Akademgorodok St., Krasnoyarsk, 660036, Russia; Deputy General Director for Research; Joint Stock Company “Novosibirsk Factory of Semiconductor Devices VOSTOK”, 60 Dachnaya St., Novosibirsk, 630082, Russia; Associate Professor, Leading Researcher; Research Institute of Internal and Preventive Medicine — Branch of the Institute of Cytology and Genetics, Siberian Branch of Russian Academy of Sciences, 175/1 B. Bogatkov St., Novosibirsk, 630089, Russia; Junior Researcher; Federal Budgetary Research Institution, State Research Center of Virology and Biotechnology “Vector”, Federal Service for Surveillance on Consumer Rights Protection and Human Wellbeing, Koltsovo, Novosibirsk Region, 630559, Russia; Design Engineer of Grade 2; ; Joint Stock Company “Novosibirsk Factory of Semiconductor Devices VOSTOK”, 60 Dachnaya St., Novosibirsk, 630082, Russia; General Director; Joint Stock Company “Design Center for Biomicroelectronic Technologies Vega”, 60a Dachnaya St., Novosibirsk, 630082, Russia

**Keywords:** biosensor, nanowire, silicon-on-insulator, field-effect transistor, troponin I, myocardial infarction, aptamer, physical adsorption

## Abstract

A biosensor based on field-effect transistors on silicon-on-insulator structures (SOI-biosensor) is a high-potential device for detection of biological molecules, for instance, such as troponin I; the biosensor allows conducting label-free real-time analysis.

**The aim of the study** is the development of SOI-biosensor design for detection of acute myocardial infarction marker — troponin I.

A notable feature of this design was the integration of two grounding electrodes directly onto the biosensor surface, which effectively nullified the static potential of the liquid sample and minimized physical breakdowns of biosensor elements.

**Materials and Methods:**

The highly specific anti-troponin I DNA aptamer was used as a receptor for specific detection of protein marker. Aptamer immobilization on the biosensor surface was carried out by physical adsorption. The analyzed range of target troponin I molecules concentration in the sample varied within 10^–11^ to 10^–9^ mol/L, mirroring clinical levels observed in myocardial infarction cases. During the experiment, a constant voltage of V_ds_=0.15 V was maintained.

**Results:**

The developed SOI-biosensor successfully detected target troponin I molecules at a concentration of 10^–11^ mol/L. The detection process exhibited an effective time of approximately 200–300 s per sample. Moreover, analysis of the detection process revealed a noticeable decrease in current within the source-drain circuit, indicative of the negatively charged complex formed by troponin I and anti-troponin I DNA-aptamer at the “liquid sample–nanowire” phase interface.

## Introduction

According to the World Health Organization, coronary heart disease (CHD) is among the major causes of death [1]. Impaired blood supply of myocardium can lead to myocardial infarction (MI) due to atherosclerotic damage to the arteries and the following necrotic processes in the heart tissue [2]. The degree of myocardium damage directly correlates with the time from the disease onset and restoration of the affected vessels patency, therefore timely and accurate diagnosis is of utmost importance for the patient prompt care.

Detection of MI-associated molecular markers is a diagnostic technique for the disease diagnosis. The major marker measured in the patient blood is troponin I (cTnI) [3]. This protein has a high clinical sensitivity to MI, as well as almost exclusive specificity to the heart tissue [4–8]. High cTnI levels in the blood flow indicate the death of myocardial contractile cells [6]. Usually, normal cTnI concentrations in the serum are less than 0.6 ng/mL (approximately 2.5×10^–11^ mol/L) [9, 10]. Minor myocardial damage can be seen at cTnI concentrations of 0.7 to 1.4 ng/ml, whereas necrotic myocardial damage is registered at protein concentrations over 1.5 ng/ml [9].

A modern high-potential diagnostic technique of troponin I detection is the use of a biosensor based on field-effect transistors on silicon-on-insulator structures (SOI-biosensor) [11–14], which allows conducting label-free real-time analysis. Moreover, it can serve as a basis for a portable tool for multicomplex analysis of various biological particles (proteins, viral particles, nucleic acids, etc.) [8, 11, 12, 15, 16].

The SOI-biosensor has two main components: a receptor layer (contains antibodies, aptamers, enzymes, etc.) and a transducer — a field-effect transistor comprises of a silicon nanowire located between the source and drain electrodes [9]. The receptor layer ensures biospecific recognition of the target molecule as the interaction of the receptor and the target molecule generates a chemical or physical signal. This signal is further converted into an electrical output signal by a transducer [8, 11, 15, 17–19]. The following SOI-biosensors are widely used: devices with a ground electrode that is introduced directly “ontop” the analyzed liquid sample to eliminate random electrical potential caused by ions and charged molecules [15, 17, 18, 20–22].

**Figure 1.**
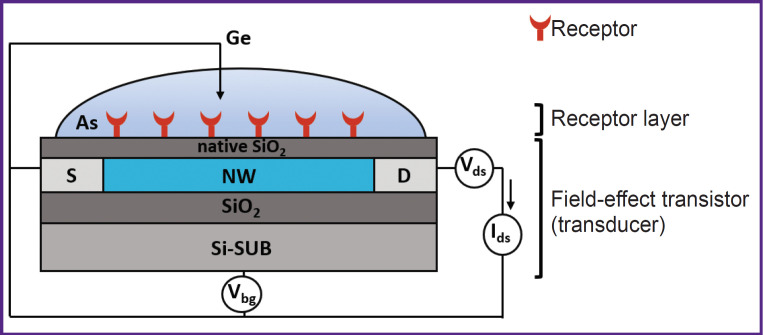
A design and electrical circuit diagram of SOI-biosensor connection [11, 13, 15, 18, 20, 23, 24]: S — the source; NW — the silicon nanowire; D — the drain; SiO_2_ — the hidden dielectric; As — the analyzed solution that contains the target molecules; Si-SUB — the silicon substrate serving as the back-gate; V_ds_ — the constant voltage source in the source-drain circuit; V_bg_ — the adjustable voltage source at the gate; I_ds_ — the ammeter; Ge — the ground electrode; native SiO_2_ — the layer of natural silicon oxide

Figure 1 shows a design and an electrical circuit diagram of SOI-biosensor connection [11, 13, 15, 18, 20, 23, 24].

Recently, aptamers were proposed as receptors [25–30]. These are short synthetic single-stranded deoxy- or ribo-oligonucleotides of a unique shape that can selectively bind to the corresponding target molecule [29–31]. Like antibodies, aptamers have high binding affinity.

The use of SOI-biosensor for detection of cTnI and other biological molecules is complicated by several issues, including the biosensor topology and design, optimal conditions for surface preparation, and the likelihood of target molecules adsorbtion to the biosensor surface [13, 15, 16, 20, 24, 32].

**The aim of the study** is the development of SOI-biosensor design for detection of the acute myocardial infarction marker — troponin I.

## Materials and Methods

### Structure of the SOI-biosensor

**Figure 2.**
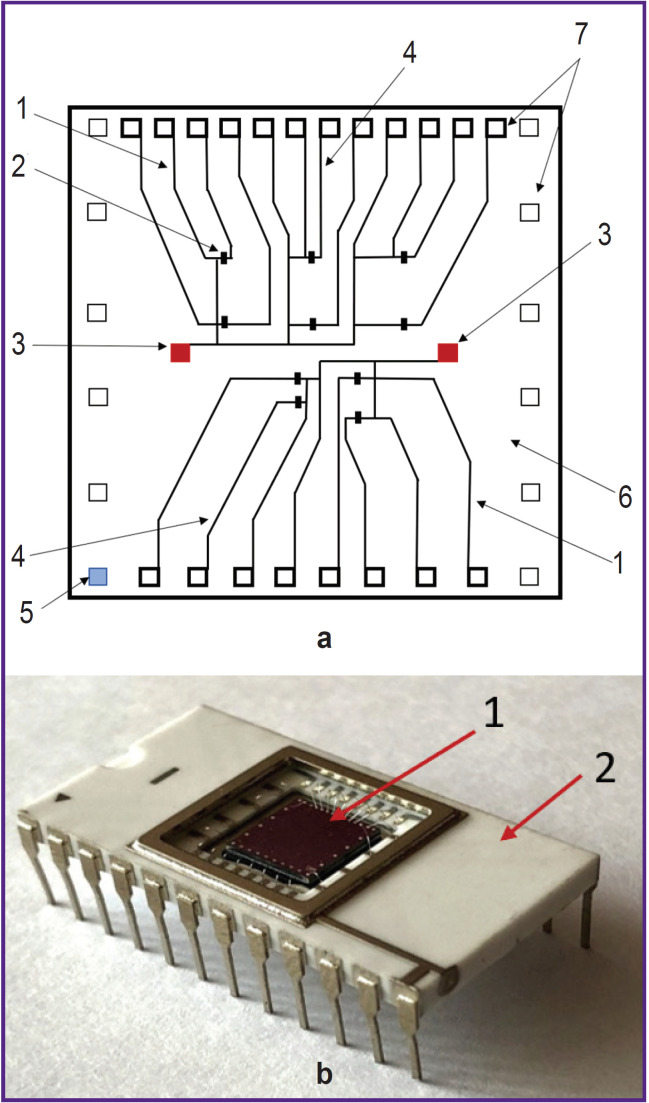
The designed device: (a) the crystal topology on the silicon-on-insulator structure: *1* — the source; *2* — the SOI-biosensor; *3* — the ground electrode; *4* — the drain; *5* — the control electrode (gate contact site); *6* — the crystal; *7* — the typical contact pads; (b) the photo of a microchip consisting of a silicon crystal with an array of ten SOI-biosensors (*1*) and a case (*2*)

**Figure 3.**
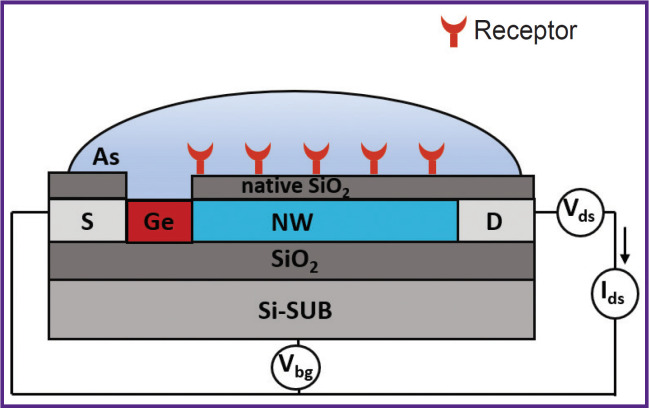
Design and the principal electrical circuit diagram of connection for the developed SOI-biosensor: S — the source; NW — the silicon nanowire; D — the drain; SiO_2_ — the hidden dielectric; As — the analyzed solution that contains the target molecules; Si-SUB — the silicon substrate serving as the back-gate; V_ds_ — the constant voltage source in the source-drain circuit; V_bg_ — the adjustable voltage source at the gate; I_ds_ – the ammeter; Ge — the ground electrode; native SiO_2_ — the layer of natural silicon oxide

The SOI-biosensor design with two integrated ground electrodes on the microchip surface was developed. The crystal topology and SOI-biosensor design are shown in Figures 2 and 3, respectively. This non-standard product was manufactured at JSC “Novosibirsk Factory of Semiconductor Devices VOSTOK” (Russia).

An array of ten SOI-biosensors with n-type conductivity was formed on a 6×6 mm silicon crystal (Figure 2 (a)). The thickness of the silicon layer (Si-SUB) was 28–30 nm, the hidden dielectric layer (SiO_2_) — 200 nm. The rear side of the crystal was attached to the microchip. Typical contact pads were connected to the microchip body with aluminum conductors. The biosensor nanowire (NW) had the following geometric characteristics: height (H) — 20 to 30 nm, width (W) — 3 μm, and length (L) — 10 μm.

One output of each SOI-biosensor was connected to the ground electrode (3) and the source (1). The second output was connected to the drain electrode — 4 (see Figure 2 (a)).

SOI-biosensors were connected to a constant voltage source — V_ds_ (see Figure 3). The biosensor operating mode was V_ds_=0.15 V.

### Materials

The following materials were used in the study: sodium chloride (NaCl), potassium chloride (KCl), disodium hydrogen phosphate (Na_2_HPO_4_), potassium dihydrogen phosphate (KH_2_PO_4_), magnesium chloride (MgCl_2_), ethanol (C_2_H_5_OH) produced by Sigma-Aldrich (USA); recombinant cardiac troponin I (cTnI) produced by HyTest (Finland).

A highly specific anti-troponin I DNA aptamer (TnAp12t2) (5’-GGAAGACAAGACATCGGGAGGGAGG GAGGGCAGTCTAGTCTCATGTGTTTCCAT GGTTC-3’) was selected by SELEX using bioluminescent reporters to monitor the DNA libraries enrichment and assess the resulting candidate sequences affinity [33]. The dissociation constant (K_D_) of the “troponin I + anti-troponin I DNA aptamer” complex was 6×10^–9^ mol/L [33].

Before the experiment, the aptamer was thermally denatured at 90°C for 5 min in the binding buffer (0.15 mol/L NaCl, 50 mmol/L K-Na phosphate buffer (pH 7.0) and 1 mmol/L MgCl_2_). Then, the aptamer was renatured at room temperature for 15 min. TnAp12t2 and cTnI solutions were diluted with the distilled water (pH 5.9) immediately before the experiment to decrease the ionic strength and conductivity of the solutions [23, 34].

### Measurement

The SOI-biosensor surface was modified to create a receptor layer by physical adsorption [35]. At the initial stage, the biosensor surface was washed with 96% ethanol and then with distilled water. Further, 5 μl of TnAp12t2 (C_A_=10^–8^ mol/L) was applied to the surface and incubated for several minutes [36]. Biosensor signals were continuously recorded (in real time) throughout the experiment. After the aptamer was added and the signal was stabilized, 5 μl of cTnI was added to the surface in the concentration of C_T_=10^–11^–10^–9^ mol/L.

The SOI-biosensor signal was a change in the current in the source–drain circuit (I_ds_) during the adsorption of biological molecules, such as aptamers or the troponin I + anti-troponin I DNA aptamer complex, onto the nanowire surface. The current was measured using the PXIe 4163 ammeter (National Instruments, USA). The voltage in the source–drain circuit (V_ds_=0.15 V) was hold constant using the PXI 4135 device (National Instruments, USA). The voltage supplied to the sensor gate was selected within V_bg_=0–30 V. The data received was visualized as the time dependence of the source– drain current — I_ds_(t).

## Results

**Figure 4.**
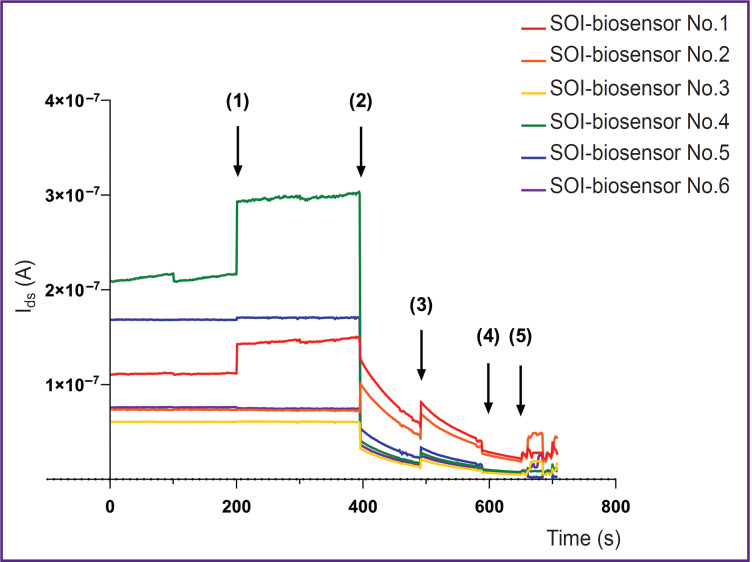
Time dependences of the source–drain current I_ds_(t) of six SOI-biosensors during the troponin I detection: (1) adding of 5 μl of 10^–8^ mol/L of the TnAp12t2 solution; (2) adding of 5 μl of 10^–11^ mol/L of the cTnI solution; (3) repeated adding of 5 μL of 10^–8^ mol/L of the TnAp12t2 solution; (4) adding of 5 μL of 10^–10^ mol/L of the cTnI solution; (5) adding of 5 μL of 10^–9^ mol/L of the cTnI solution. Voltage in the source–drain circuit V_ds_=0.15 V

The SOI-biosensor time dependences of the source– drain current I_ds_(t) are demonstrated in Figure 4. It was found that six SOI-biosensors changed the current during the experiment. The other four SOI-biosensors operated in the cut-off mode (the fully closed mode) under specified experiment conditions, thus their I_ds_(t) curves are not shown in the figure.

The initial biosensor currents differed when the devices were turned on (see Figure 4). The recorded current values covered a wide range: from I_ds_=2.1× 10^–7^ A (SOI-biosensor No.4, *green line*) to I_ds_=6.1× 10^–8^ A (SOI-biosensor No.3, *yellow line*). During the idle mode (having no biological samples on the biosensor surface) in the time range of 0–200 s, the majority of SOI-biosensors demonstrated a stable I_ds_ value, except for SOI-biosensors No.1 (*red line*) and No.4 (*green line*), which had a negligible and a noticeable current drift, respectively.

Adding 5 μl of TnAp12t2 solution (C_A_=10^–8^ mol/L) to the crystal surface at point (1) resulted in the current increase only in SOI-biosensors No.1 and No.4. Such a reaction of biosensor with the n-type conductivity was due to an increase in the number of charge carriers, here — electrons, in nanowire, indicating the presence of a positive effective electric charge at the liquid sample– nanowire phase interface. As the buffer solution had no impact on the current values, the positive charge was associated with the aptamer adsorption. Possibly under experimental conditions (pH 5.9), the positive effective electrical charge of the aptamer might be due to the protonation of adenine (pK ~3.5), cytosine (pK ~4.2), or guanine (pK ~2.1) [37–41]. The experimental results of the aptamer adsorption impact on the current value of the SOI-biosensor were consistent with the results of Farrow et al. [42].

A minor current drift was seen for SOI-biosensors No.5 (*blue line*) and No.6 (*purple line*). On the other hand, SOI-biosensors No.2 (*orange line*) and No.3 (*yellow line*) had no significant differences compared to the current in the idle mode.

Signal stabilization was recorded in the interval between points (1) and (2). However, SOI-biosensors No.1 and No.4 demonstrated a minor current drift, which indicated dynamic processes on their surface. The values of the remaining biosensors remained unchanged.

Adding 5 μl of the cTnI solution (C_1_=10^–11^ mol/L) to the crystal surface at point (2), which contained TnAp12t2 molecules, resulted in a sharp decrease in the I_ds_ values in SOI-biosensors No.1, No.3–6 in the range from 10^–1^ to 3.8 A. This was an indicator of a negative effective electric charge at the liquid sample–nanowire phase interface. As the isolated troponin molecule (pI=9.3) under the experiment conditions had a positive electrical charge, one could conclude that the resulting “troponin I + anti-troponin I DNA aptamer” complex had a negative effective electrical charge at the phase interface [42–44].

Only the I_ds_ of SOI-biosensor No.2 increased by 3×10^–8^ A. An increase in the I_ds_ values of the SOI-biosensor with n-type conductivity was possible when a positive electric charge formed on the surface of the nanowire. The results of the experiment and literature data [42–44] provide that an increase in the I_ds_ values can be caused by adsorption of the aptamer (for example, at point (1) in Figure 4) or an isolated troponin molecule (pI=9.3).

The I_ds_ values of all SOI-biosensors demonstrated a general trend to change in the time range between points (2) and (3).

At point (3), the TnAp12t2 solution (C_A_=10^–8^ mol/L) was added to the crystal surface, which resulted in the increase in current of all SOI-biosensors. A subsequent introduction of 5 μl of the cTnI solution at a concentration of C_2_=10^–10^ mol/L at point (4) led to a decrease in the I_ds_ values of all six SOI-biosensors due to formation of new “troponin I + anti-troponin I DNA aptamer” complexes. During this time range, the biosensors operated in the cut-off mode, which was aligned with their low sensitivity. Therefore, the changes in the I_ds_ values registered at points (3) and (4) were less significant than those seen at points (1) and (2).

Addition of 5 μl of the cTnI solution (C_3_=10^–9^ mol/L) at point (5) resulted in unstable operation of SOI-biosensors due to accumulation of a large number of molecules on the biosensors surface.

The spread of the I_ds_ values seen for the SOI-biosensors may be related to the chip manufacturing process, which affects biosensor sensitivity. This assumption is based on the spread of the I_ds_ values of six SOI biosensors at devices turn-on point and in the idle mode, as well as when the four SOI-biosensors operate in the cut-off mode.

## Discussion

SOI-biosensor is a high-potential analytical device for label-free detectionof the target molecules. It has high sensitivity and can record a signal in real time [11]. However, there are specific factors that complicate the SOI-biosensor application — for instance, the topology and design of the biosensor. In this study, we used a SOI-biosensor with ground electrodes located directly ontop of the microchip crystal (see Figure 2 (a)). Ten SOI-biosensors on a crystal increase the likelihood of the target molecules adsorbtion to the nanowire surface, thus improving the reliability of detection results.

An important component of effective detection with a biosensor is the availability of a receptor layer, as well as the possibility to select the type of receptors and their immobilization technique. Currently, cTnI are predominantly detected using monoclonal antibodies. An alternative approach is the application of synthetic nucleic acid sequences (aptamers). In terms of interaction specificity, aptamers are comparable to antibodies. They can be obtained using an *in vitro* evolutionary approach (SELEX) without cell lines. They can restore their activity after thermal denaturation and renaturation and exhibit high stability under tough operating conditions. Moreover, aptamers can be chemically synthesized and modified [31, 42], which makes them more cost-effective in terms of time and material costs. Aptamers can also bind to target molecules in solutions with high ionic strength, which allows the detection of proteins in undiluted biological samples such as serum, blood, etc. [23, 42]. In this study, a highly specific anti-troponin I DNA aptamer (TnAp12t2) was developed and used as a receptor; the dissociation constant (K_D_) of the “troponin I + anti-troponin I DNA aptamer” complex amounted to 6×10^–9^ mol/L. The aptamer was immobilized by physical adsorption, which allowed preserving its spatial configuration and reactivity with a simultaneous reduction of the time to prepare the biosensor for operation.

The authors of this study demonstrated the label-free real time detection of cardiac troponin I in the clinical concentration range of 10^–11^–10^–9^ mol/L using the developed design of SOI-biosensor. It was established that detection of a sample using SOI-biosensor took approximately 200 s. One should note that high concentrations of target molecules can lead to biosensor cut-off mode and incorrect results (point (5) in Figure 4). Detection of the target molecules in low concentrations is complicated by the possibility of their adsorption on the nanowire surface of SOI-biosensor. For example, adding the aptamer solution on the crystal surface at point (1) (see Figure 4) did not change the I_ds_ value of SOI-biosensors No.2, 3, 5, and 6. We assume that here aptamer molecules were not adsorbed on the nanowire surface. Thus, the likelihood of the receptor or target molecules adsorption is a key factor in detection by SOI-FET biosensor [13, 32]. A theoretical study of the target molecules detection using a SOI-biosensor is discussed in an earlier publication [45].

## Conclusion

The designed SOI-biosensor provides for real time label-free detection of troponin I. Detection of a troponin I sample using the SOI-biosensor takes approximately 200 s. The biosensor sensitivity is ~10^–11^ mol/L of protein. It was found that the aptamer immobilization by physical adsorption allowed keeping its reactivity. In the experiment, a highly specific anti-troponin I DNA aptamer (TnAp12t2) showed a positive effective electric charge at the liquid sample–nanowire phase interface. The “troponin I + anti-troponin I DNA aptamer” complex had a negative effective electrical charge at the same interface.
